# Editorial: Immunology of Vitiligo

**DOI:** 10.3389/fimmu.2021.711080

**Published:** 2021-06-24

**Authors:** Julien Seneschal, John E. Harris, I. Caroline Le Poole, Thierry Passeron, Reinhart Speeckaert, Katia Boniface

**Affiliations:** ^1^ Univ. Bordeaux, INSERM, BMGIC, U1035, Bordeaux, France; ^2^ Department of Dermatology and Pediatric Dermatology, National Reference Center for Rare Skin Disorders, Hôpital Saint-André, Bordeaux, France; ^3^ Department of Dermatology, University of Massachusetts Medical School, Worcester, MA, United States; ^4^ Departments of Dermatology, Microbiology and Immunology/Lurie Comprehensive Cancer Center, Northwestern University, Chicago, IL, United States; ^5^ INSERM U1065, Centre Méditerranéen de Médecine Moléculaire (C3M), Côte d’Azur University, Nice, France; ^6^ Department of Dermatology, CHU Nice, Côte d’Azur University, Nice, France; ^7^ Department of Dermatology, Ghent University Hospital, Ghent, Belgium

**Keywords:** vitiligo, melanocytes, innate immunity, adaptive immunity, oxidative stress, translational research, tolerance

Disappearance of melanocytes is the pathogenic hallmark of vitiligo. Progressive depigmentation of the skin has a high negative impact on patients’ quality of life. To date, vitiligo remains a therapeutic challenge (1).

Several theories have been proposed to explain disease pathogenesis, considering the roles of increased inflammatory and cytotoxic immune responses, neuropeptides, microvascular anomalies, intrinsic abnormalities in melanocyte and keratinocyte adhesion, as well as oxidative stress (2). Over the past decades, clinical, basic, and translational research on patient samples as well as *in vitro* and *in vivo* models have tremendously improved our understanding of the pathophysiology of the disease and highlighted its complexity. Such progress is of utmost importance to identify appropriate therapeutic targets and treatments to halt progression of the disease and to induce repigmentation. “Immunology of vitiligo” is a collection of six review articles and four original articles focusing on complementary aspects of the immune pathways involved in vitiligo, from a pathophysiologic to a therapeutic perspective.

Mechanisms leading to the loss of melanocytes include genetic predispositions and environmental triggers, as well as metabolic and immune alterations (3, 4). Epigenetic modifications may also be involved in vitiligo pathogenesis, as suggested in the Pu et al. study that identifies altered methylation levels of key genes involved in oxidation-reduction, inflammatory, or pigmentation processes in vitiligo melanocyte cell lines. Most of the published studies focused on the role of the immune response in non-segmental vitiligo (or vitiligo), which accounts for approximately 90% of clinical forms; segmental vitiligo being less well-studied. In their review, Speeckaert et al. emphasize the role of autoimmunity in segmental vitiligo, likely involving a targeted immune response against melanocytes carrying a somatic mutation (5). Indeed, as shown by Yang et al., transcriptomic analysis of lesional skin of segmental vitiligo versus non-segmental vitiligo showed similar changes in dysregulated immune pathways.

The innate immune response is important for the initiation of the disease. Jadeja et al. discuss the role of the endoplasmic reticulum stress-induced unfolded protein response as a bridge between oxidative stress and the development of autoimmunity in vitiligo. Danger signals (DAMPs and PAMPs) together with the role of innate immune cells in vitiligo pathogenesis is further reviewed by Boniface et al., important for subsequent activation of adaptive T cell immune responses, leading to the loss of melanocytes. Indeed, CD8+ T cells infiltrating the skin of vitiligo patients play a direct role in melanocyte disappearance, through their cytotoxic activity and the release of type 1 cytokines, in particular IFNγ and TNFα (6, 7). More recent studies also described the role of resident memory T cells expressing the IL-15 receptor, CXCR3, and NKG2D, and targeting this subset appears to prevent disease progression, flares, and to maintain repigmentation after treatment (2, 8). In this context, Plaza-Rojas and Guevara-Patiño discuss the role of the NKG2D/NKG2D ligand axis, and Willemsen et al. the relevance of targeting the PD-1/PD-L1 axis in vitiligo. Disruption of tolerance is a hallmark of autoimmunity and previous studies describe dysregulation of regulatory T cell function and/or number in patients (9, 10). In their original article, Mukhatayev et al. elegantly investigated the potential of using CAR Tregs as a therapeutic strategy in a pre-clinical humanized mouse model prone to develop vitiligo. The importance of using appropriate *in vitro* and *in vivo* pre-clinical models to perform mechanistic and translational studies is outlined in the Katz and Harris review. Animal models with spontaneous development of vitiligo are not optimal to fully understand the complexity of the human disease. In their case series, Egbeto et al. described a similar immune profile between canine and human autoimmune pigmentary disorders, suggesting that findings in one model could be relevant to the other.

In conclusion, the prominent role of autoimmunity in vitiligo pathogenesis is now evident, as exemplified with recent clinical studies evaluating the efficacy of drugs targeting the immune response, such as JAK inhibitors (11, 12). Yet prediction of drug efficacy is important for personalized therapeutic management of patients. Yang et al. identified biomarkers of innate and adaptive immune responses associated with favorable response to therapy. It will be also important to assess whether new therapies induce durable repigmentation of vitiligo, or whether maintenance therapies will be required to prevent disease relapse.

## Author Contributions

JS, JH, IP, TP, RS, and KB wrote the editorial and invited authors to participate in the collection. All authors contributed to the article and approved the submitted version.

## Conflict of Interest

JH is a Scientific Founder of Villaris Therapeutics, Inc. JS and KB received financial support from Sanofi Genzyme and Calypso Biotech that is separate from this proposed editorial. IP is the CSO for Temprian Therapeutics, a company hoping to bring an HSP70iQ435A-based treatment to clinical trials for vitiligo. The approach for this treatment is different and separate from the proposed Research Topic.

The remaining authors declare that the research was conducted in the absence of any commercial or financial relationships that could be construed as a potential conflict of interest.

## References

[1] Ezzedine K Eleftheriadou V Whitton M van Geel N. Vitiligo. Lancet (2015) 386:74–84. 10.1016/S0140-6736(14)60763-7 25596811

[2] Frisoli ML Essien K Harris JE. Vitiligo: Mechanisms of Pathogenesis and Treatment. Annu Rev Immunol (2020) 38:621–48. 10.1146/annurev-immunol-100919-023531 32017656

[3] Picardo M Dell’Anna ML Ezzedine K Hamzavi I Harris JE Parsad D. Vitiligo. Nat Rev Dis Primers (2015) 1:15011. 10.1038/nrdp.2015.11 27189851

[4] Boniface K Seneschal J Picardo M Taïeb A. Vitiligo: Focus on Clinical Aspects, Immunopathogenesis, and Therapy. Clin Rev Allergy Immunol (2018) 54:52–67. 10.1007/s12016-017-8622-7 28685247

[5] van Geel N Mollet I Brochez L Dutré M De Schepper S Verhaeghe E. New Insights in Segmental Vitiligo: Case Report and Review of Theories. Br J Dermatol (2012) 166:240–6. 10.1111/j.1365-2133.2011.10650.x 21936857

[6] Boniface K Seneschal J. Vitiligo as a Skin Memory Disease: The Need for Early Intervention With Immunomodulating Agents and a Maintenance Therapy to Target Resident Memory T Cells. Exp Dermatol (2019) 28:656–61. 10.1111/exd.13879 30636075

[7] Riding RL Harris JE. The Role of Memory Cd8+ T Cells in Vitiligo. J Immunol (2019) 203:11–9. 10.4049/jimmunol.1900027 PMC6709670 31209143

[8] Seneschal J Boniface K D’Arino A Picardo M. An Update on Vitiligo Pathogenesis. Pigment Cell Melanoma Res (2021) 34:236–43. 10.1111/pcmr.12949 33278065

[9] Dwivedi M Kemp EH Laddha NC Mansuri MS Weetman AP Begum R. Regulatory T Cells in Vitiligo: Implications for Pathogenesis and Therapeutics. Autoimmun Rev (2015) 14:49–56. 10.1016/j.autrev.2014.10.002 25308528

[10] Mukhatayev Z Ostapchuk YO Fang D Le Poole IC. Engineered Antigen-Specific Regulatory T Cells for Autoimmune Skin Conditions. Autoimmun Rev (2021) 20:102761. 10.1016/j.autrev.2021.102761 33476816 PMC9285649

[11] Rosmarin D Pandya AG Lebwohl M Grimes P Hamzavi I Gottlieb AB. Ruxolitinib Cream for Treatment of Vitiligo: A Randomised, Controlled, Phase 2 Trial. Lancet (2020) 396:110–20. 10.1016/S0140-6736(20)30609-7 32653055

[12] Passeron T. First Step in a New Era for Treatment of Patients With Vitiligo. Lancet (2020) 396:74–5. 10.1016/S0140-6736(20)30747-9 32653058

